# Relationship Between Substance Use and Suicide Behavior During the COVID-19 Pandemic: A Systematic Review and Random-Effects Proportions Meta-Analysis

**DOI:** 10.3390/jcm15041328

**Published:** 2026-02-07

**Authors:** Estefano D. Cadena Barberis, Ha Ram Oh, Luis David Vélez Ordóñez, Valeria Salomé Calvopiña, Jose A. Rodas, Jose E. Leon-Rojas

**Affiliations:** 1 NeurALL Research Group, Quito 170157, Ecuador; estefano.cadena@udla.edu.ec (E.D.C.B.);; 2 Escuela de Medicina, Universidad de Las Américas, Quito 170124, Ecuador; ha.oh@udla.edu.ec; 3 School of Psychology, University College Dublin, D04 V1W8 Dublin, Ireland; 4 Escuela de Psicología, Universidad Espíritu Santo, Samborondón 092301, Ecuador; 5 Grupo de Investigación Bienestar, Salud y Sociedad, Escuela de Psicología y Educación, Universidad de Las Américas, Quito 170124, Ecuador

**Keywords:** COVID-19 pandemic, substance use, suicidal behavior, suicidal ideation, proportions meta-analysis

## Abstract

**Background/Objectives:** The COVID-19 pandemic disrupted social structures, healthcare access, and psychological well-being, potentially intensifying substance use and suicidal behavior. Although both phenomena have been independently studied, their co-occurrence during the pandemic has not been systematically synthesized. To evaluate the prevalence and patterns of suicidal behavior among individuals with substance use during the COVID-19 pandemic through a systematic review and random-effects proportions meta-analysis. **Methods:** A systematic search of PubMed, Scopus, Web of Science, and EBSCO Host was conducted from 11 March 2020 to 15 October 2022 for studies published between March 2020 and October 2022. Eligible studies included observational designs reporting substance use and suicidal behavior in adults during the pandemic. Risk of bias was assessed using National Institutes of Health tools. Proportional meta-analyses were performed using a random-effects model with Freeman–Tukey double arcsine transformation. Heterogeneity was quantified using the I^2^ statistic. **Results:** Twenty studies comprising 70,684 individuals were included. Substance use during the pandemic was reported in 24.6 percent of participants, while 30.7 percent exhibited suicidal behavior. A total of 16.1 percent presented with both substance use and suicidal behavior. The pooled prevalence of any suicidal behavior among individuals with substance use was 33.8 percent (95 percent CI, 22.8 to 45.7), with substantial heterogeneity. Alcohol showed a pooled prevalence of 36.2 percent, cannabis 48.1 percent, and tobacco 11.5 percent. Suicidal ideation was the most frequent outcome, with a pooled prevalence of 36.8 percent among substance users. Most studies reported an increased association between substance use and suicidal behavior compared with pre-pandemic periods. **Conclusions:** Substance use and suicidal behavior frequently co-occurred during the COVID-19 pandemic, particularly suicidal ideation and alcohol use. These findings highlight the need for integrated mental health and substance use interventions during public health crises.

## 1. Introduction

The relationship between substance use and suicidal behavior gained significant attention during the COVID-19 pandemic, particularly because substance abuse could act as a potential coping mechanism in a moment of uncertainty, social isolation, and heightened stress levels [1,2,3]. Substance use has been previously identified as a maladaptive coping mechanism, often resulting in addiction, negative behavioral changes, and, in some cases, withdrawal syndrome that develops particularly during periods of restricted access, such as those caused by pandemic-related policies [4,5]. Furthermore, substance use has been reported as a significant contributor to suicidal ideation and behavior, as it can exacerbate underlying mental health conditions and intensify feelings of hopelessness and despair [6,7,8,9].

Beyond acute stressors, the COVID-19 pandemic introduced sustained disruptions to social structures, healthcare access, employment, and daily routines, all of which are recognized determinants of mental health and suicide risk [2,3,6]. Lockdowns, quarantine measures, and physical distancing policies reduced access to protective social networks while simultaneously increasing exposure to psychological stress, loneliness, and economic uncertainty; these conditions disproportionately affected individuals with pre-existing psychiatric disorders, substance use disorders, and socioeconomic vulnerabilities, worsening both substance consumption patterns and suicide-related outcomes [2,3,6]. Emerging evidence suggests that changes in substance use during the pandemic were not uniform but varied according to substance type, demographic characteristics, and regional policy responses [6,7]. Alcohol consumption, misuse of prescription medications, and illicit drug use were reported to increase in specific populations, particularly among individuals experiencing anxiety, depression, or chronic stress [6,7]. These patterns are clinically important, as both acute intoxication and chronic substance use are associated with impaired judgment, emotional dysregulation, and increased impulsivity, all of which are established proximal risk factors for suicidal behavior [6,7]. Importantly, the pandemic context also altered access to mental health and addiction services. Disruptions in outpatient care, reduced availability of interventions, and delays in emergency psychiatric evaluation may have contributed to unmet treatment needs [1,2,5]. Additionally, increased reliance on self-medication with psychoactive substances may have further worsened psychiatric symptoms and increased vulnerability to suicidal ideation and attempts; these systemic factors highlight the need to examine substance use and suicide risk within a broader public health framework [1,2,5]. Moreover, it is important to note that the COVID-19 pandemic did not represent a uniform exposure across countries or regions; certainly, public health responses varied widely with respect to the timing, duration, and severity of restrictions, including lockdowns, quarantine measures, access to healthcare services, educational disruptions, and limitations on social interaction. These policy-driven differences likely influenced patterns of psychoactive substance use, help-seeking behavior, and suicide risk in context-specific ways. Consequently, the associations described in this review should be interpreted within the framework of heterogeneous regional responses to the pandemic, rather than as effects of a single, standardized global intervention.

Although the acute phase of the COVID-19 pandemic has ended, its mental health consequences have not; the pandemic functioned as a prolonged, population-wide stressor that intensified substance use behaviors, disrupted access to mental health and addiction services, and exacerbated suicide risk factors [6,7]. Emerging evidence suggests that increases in psychoactive substance use, delayed psychological distress, and unmet treatment needs have persisted beyond the lifting of public health restrictions [2,3,6,7]. As such, examining the relationship between substance use and suicidal behavior during the pandemic remains highly relevant, not as a retrospective exercise, but as a means of understanding the downstream and potentially enduring effects of large-scale societal crises. Insights derived from this period may inform post-pandemic suicide prevention strategies, addiction services planning, and preparedness for future public health emergencies.

Even though individual studies have explored substance use or suicidal behavior during the COVID-19 pandemic, findings remain highly heterogeneous, with variability in study design, populations, substances examined, and outcome definitions. Moreover, the extent to which substance use specifically mediated or moderated suicidal ideation and behavior during this period has not been comprehensively synthesized. Therefore, a systematic evaluation of the available evidence is necessary to clarify these associations, identify high-risk groups, and inform prevention strategies during future large-scale public health crises; which is why we decided to evaluate substance use, its effects, and its relation to suicidal ideation and behavior during the COVID-19 pandemic through the means of a systematic methodology that implements a random-effects proportions meta-analysis where feasible.

## 2. Materials and Methods

This systematic review and meta-analysis were conducted according to the Preferred Reporting Items for Systematic Reviews and Meta-Analysis (Supplementary Materials PRISMA) guidelines, including the registry of our research protocol in the International Prospective Register of Systematic Reviews (PROSPERO) under the ID CRD42023421552.

### 2.1. Eligibility Criteria

#### 2.1.1. Study Types

We included cross-sectional, cohort, and case–control studies that mentioned the relationship between substance use-related disorders and the development of suicidal ideation or behavior during the pandemic. All secondary studies (systematic, scoping, and narrative reviews) and those not based on humans were excluded.

#### 2.1.2. Participants

Subjects from all ages, genders, cultures, and countries were included, as long as they had a clear diagnosis of substance abuse as well as a type of suicidal behavior; for the purpose of this study, the definition of substance abuse provided by the United States Centers for Disease Control and Prevention (CDC) was considered: “Use of selected substances, including alcohol, tobacco products, illicit drugs, inhalants, and other substances that can be consumed, inhaled, injected, or otherwise absorbed into the body with possible dependence and other detrimental effects”. On the other hand, the definition of suicide behavior is heterogeneous, as not all considered papers were clear as to what they considered to be a suicide behavior. However, for the purpose of our review, it is defined as a spectrum of behaviors from suicide risk and/or preparatory behaviors, attempts up to completed suicide that may or may not include suicidal ideation.

#### 2.1.3. Language

We included articles in Spanish, English, Korean, French, or Italian. All articles conducted with the aforementioned criteria and conducted during and/or analyzing the effect of the COVID-19 pandemic.

### 2.2. Information Sources and Search Strategy

An electronic search was conducted in four databases: Scopus, PubMed, Web of Science, and EBSCO host from 11 March 2020 to 15 October 2022, using a combination of free-text and medical subheadings (MeSH) terms that considered the following key terms: COVID-19, substance use (abuse), suicide, suicide behavior; no restrictions or filters were applied and all databases were queried from 11 March 2020 to 15 October 2022 considering the COVID-19 onset, its lockdown periods, its peak, and its subsequent diminishment as COVID-19 pandemic measures were lifted and vaccination efforts progressed worldwide. Studies in English, Spanish, French, Italian, and Korean were included, addressing all primary studies analyzing the relationship between substance use and the development of suicidal behavior during the pandemic. In each database, keywords and their variants were used to identify articles related to suicide, substances, and COVID-19.

### 2.3. Selection Process

Mendeley Desktop (version 1.19.8) was used during the data selection process to identify and remove duplicate entries, ensuring an accurate count of unique articles for the initial screening. Two reviewers (S.C. and D.O.) independently assessed titles and abstracts according to predefined inclusion criteria. Discrepancies were resolved by consensus and with the participation of a third reviewer (E.C.). To ensure methodological rigor, a second full-text review was performed on articles that passed the initial screening; this review focused on studies examining the association between substance use disorders, specifically involving substances with abuse potential as classified by the National Institute on Drug Abuse (NIDA), and the emergence of suicidal behavior during the COVID-19 pandemic. Four reviewers conducted blind, independent reviews, and any disagreements were solved by mutual consensus before data extraction and bias assessment.

### 2.4. Data Items

Data collection was carried out by creating a database in Microsoft Excel and manually extracting the information of each article that was selected; variables considered included aspects related to the different suicidal behaviors and substance use, basic article information (author, year, DOI, study design), demographic data (age, gender), type of substance and usage dimension, the relationship between substance use and suicidal ideation, and the impact of the COVID-19 pandemic, if specified. For the meta-analysis, specific absolute frequencies for those diagnosed with substance abuse and who presented suicidal behavior were extracted and compared with the total population in order to calculate a prevalence.

### 2.5. Risk of Bias

The bias analysis was performed using the study quality assessment tools from the National Institutes of Health. The bias of each article was independently reviewed by two researchers, and any discrepancies were resolved by a third through mutual consensus. To evaluate the risk of bias, two researchers independently read the articles included and applied a questionnaire of 14 questions, based on the study design, and classified them into low, moderate, and high risk of bias based on the percentage of positive (yes) answers as follows: ≥80% (low risk), 50–79% (moderate risk), and <50% (high risk).

### 2.6. Data Synthesis and Analysis

Two primary outcomes were defined for this review. The first was the presence or prevalence of suicidal ideation or suicidal behavior, including suicide attempts, among individuals with reported substance use during the COVID-19 pandemic. The second outcome concerned the relative distribution of substance use types associated with suicidal outcomes, such as alcohol, illicit drugs, or non-medical use of prescription medications. The primary outcome was used to perform a proportional meta-analysis employing a random-effects model with the Freeman–Tukey double arcsine transformation to stabilize variance; pooled estimates were reported as proportions with corresponding 95% confidence intervals. Statistical heterogeneity was quantified using the I^2^ statistic and interpreted as low (I^2^ < 25%), moderate (I^2^ = 25–74%), or high (I^2^ ≥ 75%). In instances of substantial heterogeneity, sensitivity analyses were conducted to explore potential sources contributing to between-study variability; assessment of publication bias was conducted qualitatively and incorporated into the overall risk-of-bias evaluation, as conventional quantitative approaches such as funnel plots and Egger regression are not appropriate for meta-analyses of proportions and may yield misleading results in this context. All statistical analyses were performed using the Joanna Briggs Institute System for the Unified Management, Assessment and Review of Information (JBI SUMARI), Adelaide, Australia.

Given the anticipated clinical, methodological, and contextual heterogeneity across studies conducted during different phases of the COVID-19 pandemic and in diverse populations, pooled prevalence estimates were interpreted as descriptive summaries rather than precise population parameters. In this context, the purpose of the random-effects meta-analysis was not to derive a single generalizable prevalence estimate, but to characterize the overall magnitude and variability of co-occurring substance use and suicidal behavior across heterogeneous settings. Accordingly, pooled estimates should be interpreted alongside heterogeneity metrics and study-level distributions, recognizing that high I^2^ values reflect genuine between-study variability rather than solely statistical imprecision.

## 3. Results

### 3.1. Study Selection

**Figure 1:**
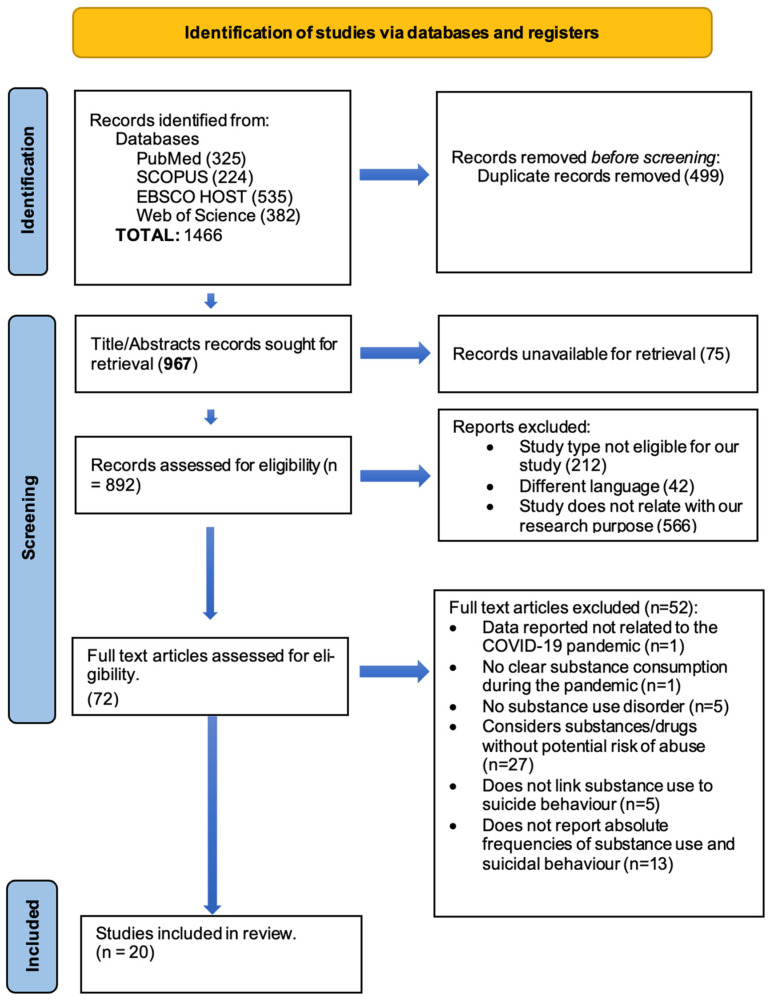
PRISMA flow diagram of the study selection process.

After an initial analysis of 1466 abstracts, 967 were considered after duplicate deletion; from these, 75 reports were not available for retrieval. Therefore, 892 studies went through our full screening process, resulting in a total of 20 articles selected for our study [10,11,12,13,14,15,16,17,18,19,20,21,22,23,24,25,26,27,28,29]. The full selection process is presented in the PRISMA flow diagram (Figure 1).

### 3.2. Risk of Bias

Out of the 20 articles that were included for this study, 2 articles scored an estimate of 11 out of 14 positive responses, resulting in a low risk of bias; 11 articles scored between 7 and 11 positive responses, resulting in a moderate risk of bias; and 7 articles scored lower than 7 positive responses, resulting in a high risk of bias. Most of the articles that were considered as high risk of bias were unable to meet rigorous methodological standards due to the inherent difficulties of conducting research in the context of a world pandemic, including issues with follow-up and obtaining objective measures of exposure and outcomes.

### 3.3. Substance Abuse and Suicide Behavior During the COVID-19 Pandemic

**Figure 2:**
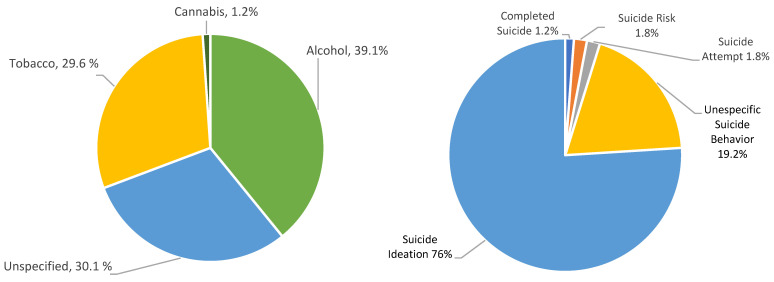
Distribution of calculated relative frequencies of reported substances and suicide behaviors among individuals with concurrent substance use and suicidal behavior during the COVID-19 pandemic.

A total of 70,684 cases were considered from the 20 included research articles [10,11,12,13,14,15,16,17,18,19,20,21,22,23,24,25,26,27,28,29]. Among these, 17,384 (24.6%) reported substance use during the pandemic, and 21,663 (30.7%) reported a form of suicidal behavior (suicidal ideation, suicidal risk, completed suicide, attempted suicide, and/or unspecified suicide behavior) [10,11,12,13,14,15,16,17,18,19,20,21,22,23,24,25,26,27,28,29]. Within the total population, 11,382 (16.1%) represented individuals that concurrently presented with substance use and any type of suicidal behavior, as shown in Table 1 [10,11,12,13,14,15,16,17,18,19,20,21,22,23,24,25,26,27,28,29]. Among these, the main substances reported were alcohol in 4453 cases, unspecified substances in 3435 cases, tobacco in 3372 cases, and cannabis in 122 cases; conversely, among the main suicidal behaviors in substance users, suicidal ideation was the most frequent, reported in 8646 cases, followed by unspecified suicidal behavior in 2187 [10,11,12,13,14,15,16,17,18,19,20,21,22,23,24,25,26,27,28,29]. The relative frequencies of both substances and suicidal behavior are presented in Figure 2.

For our proportions random-effects meta-analysis, we conducted a series of evaluations looking into all substances and all suicide behaviors, as well as specific substances with suicide behaviors, and suicidal ideation (the most common behavior) with all substances, with sensitivity analysis (removal of outliers). The main caveat of our analyses is the significant heterogeneity observed even after sensitivity analysis, with a calculated I^2^ statistic ranging from 99.8 to 74.2 (i.e., a high degree of heterogeneity). Therefore, we must exert caution during the interpretation of these results and their application in general; furthermore, this degree of heterogeneity showcases the difficulty in assessing such a complex association as suicide behavior and substance abuse during a global pandemic, which affected the execution of the studies due to problems in follow-up and recruitment of participants.

**Figure 3:**
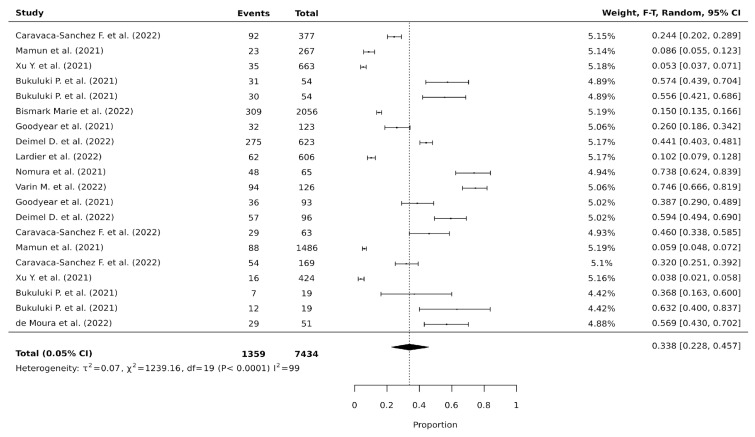
Forest plot of the random-effects proportions meta-analysis of all substances and all suicide behaviors after sensitivity analysis [11,14,15,16,17,19,20,22,23,24,29].

When looking into all substances and all suicide behaviors, after sensitivity analysis performed by removing 7 articles [10,12,18,21,26,27,28] from specific comparisons that reported a proportion of 100% between a determined substance and suicide behavior, we calculated a pooled prevalence of 33.8% (95% CI, 22.8–45.7, *p* < 0.0001; I^2^ = 99.0) of any suicide behavior in those with a reported substance abuse during the COVID-19 pandemic (Figure 3).

**Figure 4:**
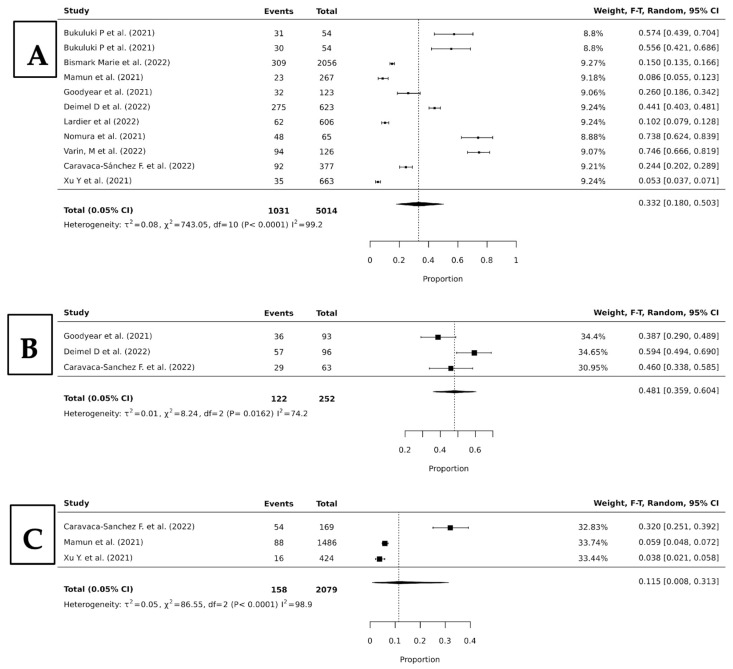
Forest plots of proportion random-effects meta-analyses looking at the pooled prevalence of alcohol (**A**), cannabis (**B**), and tobacco (**C**) abusers who presented with any suicide behavior [11,14,15,16,17,19,20,22,24,29].

When looking at specific substances and their influence in all suicide behavior, we found a pooled any-suicide-behavior prevalence of 36.2% (95% CI, 17.7–57.1; *p* < 0.0001; I^2^ = 99.3), 48.1% (95% CI 35.9–60.4; *p* = 0.0162; I^2^ = 74.2), and 11.5% (95% CI, 0.8–31.3; *p* < 0.0001; I^2^ = 98.9) for alcohol, cannabis, and tobacco, respectively (Figure 4).

**Figure 5:**
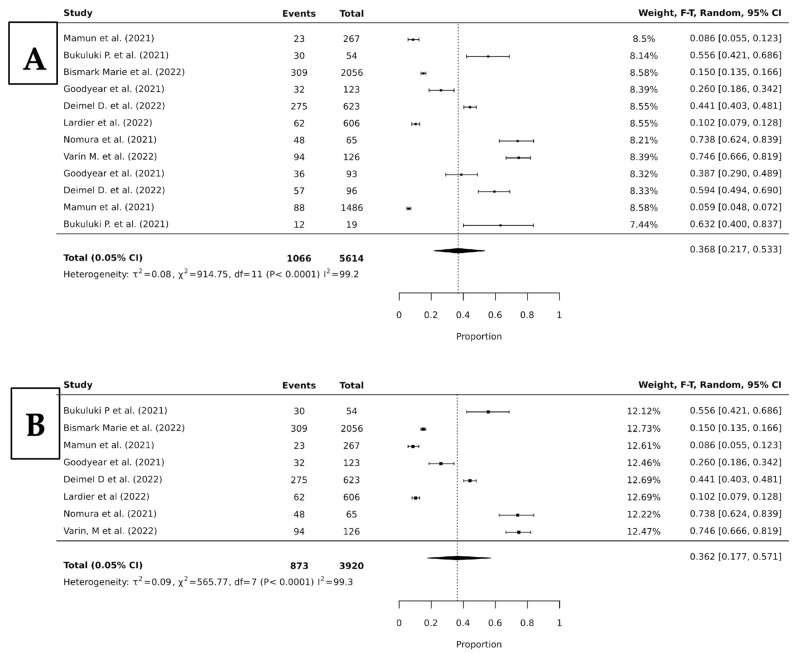
Forest plots of proportion random-effects meta-analyses looking at the pooled prevalence of any drug (**A**), and alcohol (**B**) with suicide ideation [11,14,15,16,17,19,20,22].

The last meta-analysis looked into the most common type of suicide behavior (suicide ideation) and the prevalence of co-occurrence with all drugs and just alcohol resulting in a pooled prevalence of 36.8% (95% CI, 21.7–53.3; *p* < 0.0001; I^2^ = 99.2), and 36.2% (95% CI 17.7–57.1; *p* < 0.0001; I^2^ = 99.3), respectively (Figure 5).

Finally, to determine the influence of COVID-19 on the population with concurrent substance use and suicide behavior, a qualitative conjoined analysis was performed for each article to evaluate whether the association between these two factors was reported to have increased, decreased, or remained unchanged, compared to pre-pandemic times. Out of the 4453 alcohol users with concurrent suicide behavior, 95.9% reported an increased association, 0.4% a decreased association, and 3.7% an unchanged association; of the 3435 undetermined substance users with suicide behavior, 99.9% demonstrated an increased association, while 0.1% a decreased association [10,11,12,13,14,15,16,17,18,19,20,21,22,23,24,25,26,27,28,29]. Finally, out of the 122 reported cannabis users, 53.3% reported an increased association while 46.7% reported an unchanged association to any suicide behavior [10,11,12,13,14,15,16,17,18,19,20,21,22,23,24,25,26,27,28,29].

## 4. Discussion

COVID-19 exposed multiple failings in healthcare and public health inequities all over the world; the pandemic allowed us to look into significant shortcomings related to health access but also related to the dynamics of the pandemic itself (i.e., isolation, social distancing, mobility restrictions, etc.). Such dynamics have been studied as factors associated with multiple health conditions that worsened or were heightened during the pandemic, such as mental health issues and substance abuse [1,2,3,4,5,6,7,8,9]. In this systematic review, we decided to focus on the relationship between substance abuse during the pandemic and the appearance of suicidal behavior due to the context of the pandemic itself. We found that, out of 70,684 individuals included in our review, originating from 20 research articles [10,11,12,13,14,15,16,17,18,19,20,21,22,23,24,25,26,27,28,29], 24.6% reported substance use during the pandemic, and 30.7% reported a form of suicidal behavior. When focusing on those individuals that presented both occurrences (i.e., substance use and suicidal behavior) through the means of a random-effects proportions meta-analysis, we found a pooled prevalence of 33.8% (95% CI, 22.8–45.7, *p* < 0.0001; I^2^ = 99.0), with cannabis and alcohol showing pooled prevalences of 48.1% (95% CI 35.9–60.4; *p* = 0.0162; I^2^ = 74.2) and 36.2% (95% CI, 17.7–57.1; *p* < 0.0001; I^2^ = 99.3), respectively [10,11,12,13,14,15,16,17,18,19,20,21,22,23,24,25,26,27,28,29]. Furthermore, suicidal ideation was the most common behavior, with a pooled prevalence in any drug use of 36.8% (95% CI, 21.7–53.3; *p* < 0.0001; I^2^ = 99.2) [10,11,12,13,14,15,16,17,18,19,20,21,22,23,24,25,26,27,28,29]. However, a key consideration when interpreting our findings is the consistently high heterogeneity observed across all pooled analyses. I^2^ values approaching 99% indicate that the variability in prevalence estimates is largely driven by real differences between studies rather than random error. This heterogeneity likely reflects substantial variation in study populations, pandemic phases, public health restrictions, healthcare access, substance availability, and definitions of both substance use and suicidal behavior. Under such conditions, pooled prevalence estimates should not be interpreted as definitive or universally applicable values. Instead, they provide a quantitative synthesis that illustrates the breadth and magnitude of co-occurrence across highly diverse contexts. In this sense, the meta-analysis serves to demonstrate that the co-occurrence of substance use and suicidal behavior during the COVID-19 pandemic was common and substantial, while simultaneously underscoring that its expression was strongly context dependent.

First, it should be noted that this study does not determine a causal relationship but instead aims to explore the impact of the COVID-19 pandemic on both of these multifactorial conditions. The association between substance use and suicidal behavior has been consistently documented in the scientific literature, even before the pandemic. Conner et al., reported in their meta-analysis that substance use could significantly increase suicide risk (OR = 7.18, CI 95%: 3.22–16.01), being the most common disorder alcohol abuse (OR = 3.94, CI 95%: 2.04–7.59); meanwhile, cannabis use and suicide behavior did not revealed any correlation (OR = 2.51, CI 95%: 0.60–10.54) [30]. These results align with our study, as we found that the main substance studied in suicide behavior was alcohol in 14 out of 20 studies, in 4273 subjects out of 8382 alcohol users. In contrast, our findings for cannabis use differed, as two out of the three studies that considered cannabis use found an increased association for suicide behavior for 122 individuals out of 252 cannabis users and a pooled prevalence of 48.1% (higher than the alcohol pooled prevalence). Considering tobacco use, we found a pooled prevalence of 11.5% (95% CI, 0.8–31.3; *p* < 0.0001; I^2^ = 98.9) with any suicide behavior but found an overall 29.6% consumption reported in the literature. This finding may reflect a maladaptive coping mechanism previously described by Bindu et al., where stress was found to be a major factor in both initiation and maintenance of smoking, as it presented to have a clear relation for 70% of smokers [31]. Nonetheless, the SARS-CoV-2 pandemic constituted a stress factor on its own right, where 13 out of 20 studies reviewed reported an influence on suicide behavior and 11 out of 20 an influence on substance use. These effects add to the numerous factors the pandemic introduced; from its onset, uncertainty regarding symptomatology and mortality risk generated widespread fear, prompting the implementation of public health measures, including social distancing and lockdowns, which, in turn, disrupted daily routines, reduced access to social support, had economic ramifications, and led to loss of loved ones [32].

Thus, the pandemic increased mental burden on the general population, with certain subgroups disproportionately affected. Nomura et al. reported that weekly alcohol consumption in enrolled university students was associated with depressive symptoms and increased suicide behavior (OR = 2.60, 95% CI, 1.03–6.55) [20]. Certainly, alcohol consumption by students amidst an uncertain future and possible unemployment has a positive association with suicidal ideation [21]. The lockdown itself, without any protective factors such as exercise or good relationships among family members, resulted in substance use as an instrument to dissociate from reality [21]. Nonetheless, the demographics in our recollected data suggests that patients who had suicidal ideation or behaviors during the inception of the pandemic were mostly individuals who had worsened mental health due to persistent anxiety, stress, and depressive events. As Goodyear et al. suggest, substance use with suicide ideation was highly prevalent in their sample study among LGBTQ2 individuals: 2.28 (95% CI, 1.28–4.07); as a result of the mental state, individuals coped with mostly alcohol by approximately 30% and 19% with cannabis use [16].

Regarding the type of suicide behavior during the COVID-19 pandemic, our study found that the main type of suicide behavior reported in the literature was suicide ideation (75.96%), sharing a comparable tendency to Poorolajal et al. pre-pandemic meta-analysis where substance use disorder doubled the odds of suicide ideation, however in contrast to their findings, our study did not demonstrate a similarly strong association between substance use and suicide attempt OR = 2.49 (95% CI: 2.00–2.98) [6]. This may result from pandemic measures, such as lockdowns that, in certain subgroups, allowed greater family presence, and in others, reduced access to suicidal means, including the progress of telemedicine during recent years, allowing for better treatment options and mental burden management [33].

### Study Limitations

Our systematic review has several important limitations that should be carefully considered when interpreting its findings. First, a substantial proportion of the included studies did not clearly specify either the type of suicidal behavior or the exact substance involved. Although this limitation reflects the real-world complexity of clinical and epidemiological reporting during a global health emergency, it introduced unavoidable classification ambiguity. To mitigate this issue, we created predefined categories for unspecified suicidal behavior and unspecified substances. However, due to their intrinsic heterogeneity and the risk of misinterpretation, these categories were excluded from substance-specific inferential discussions, which may have led to an underestimation of certain associations. Second, the included studies were conducted across heterogeneous sociocultural contexts and pandemic phases, characterized by marked variability in lockdown severity, duration, healthcare accessibility, and public health responses. This contextual diversity represents a major source of confounding and limits direct cross-study comparability. Also, the wide heterogeneity observed in all pooled analyses, even after sensitivity testing, underscores the multifactorial nature of suicidal behavior and substance use during the pandemic and restricts the generalizability of pooled prevalence estimates. Third, most studies relied on cross-sectional designs and self-reported data, precluding causal inference and increasing susceptibility to recall and reporting biases. Additionally, few studies explicitly examined substance withdrawal or disrupted access to substances during lockdowns, despite withdrawal syndromes being clinically recognized precipitants of mood instability and suicidality. This gap highlights an important blind spot in the existing literature. Despite these limitations, our review provides a comprehensive synthesis of the available evidence and underscores the urgent need for standardized definitions, outcome reporting, and longitudinal designs to better determine causal pathways linking substance use and suicidal behavior during large-scale public health crises.

## 5. Conclusions

Our systematic review and random-effects proportions meta-analysis shows the existence of a co-occurrence of substance use and suicidal behavior during the COVID-19 pandemic (albeit with important heterogeneity). Approximately one-third of individuals with substance use reported some form of suicidal behavior, with suicidal ideation emerging as the predominant manifestation. Alcohol was the substance most frequently associated with suicidal outcomes, followed by cannabis and tobacco, although the former showed greater heterogeneity across studies. Our findings indicate that the pandemic did not merely coexist with these phenomena but could have amplified them through social isolation, psychological distress, disrupted healthcare access, and economic instability. Vulnerable populations, including university students, healthcare workers, and individuals suffering from mental health issues, could have been disproportionately affected, reinforcing the role of substance use as a maladaptive coping strategy under prolonged stress conditions. Therefore, during large-scale crises, substance use and suicidal behavior should be addressed as interconnected public health emergencies rather than isolated outcomes. Prevention strategies should integrate suicide risk assessment into substance use services, account for withdrawal and access disruptions, and take advantage of telemedicine and digital interventions to maintain continuity of mental health care. Failure to adopt integrated and anticipatory approaches risks replicating the same vulnerabilities in future pandemics or global emergencies.

## Figures and Tables

**Table 1:** Reported associations between substance use and suicide behavior by substance type and population during the COVID-19 pandemic.

Author	Year	Country	Target Population	(*n*) Total Population of the Study During the COVID-19 Pandemic	Age in Years	Sex	(*n*) Suicide Behavior with or Without Substance Use During the COVID-19 Pandemic	Type of Substance	(*n*) Who Used Substance(s) During the COVID-19 Pandemic	% of the Total Population Who Used Substance(s) During the COVID-19 Pandemic	Suicide Behavior	(*N*) Who Used Substance(s) and Had a Suicide Behavior During the COVID-19 Pandemic	% of the Population Who Used Substance(s), Who Had a Suicide Behavior During the COVID-19 Pandemic
Gerstner R et al. [10]	2022	Ecuador	Individuals who completed suicide in Ecuador	1593	≥15	Male: 78.7% Female: 21.3%	1593	Alcohol	139	8.72	Completed Suicide	139	100
Bukuluki P et al. [11]	2021	Uganda	Adolescents receiving services from Uganda Youth Development Link during COVID-19	219	13–19	Male: 32% Female: 68%	101	Alcohol	54	23.1	Suicide Attempt	31	57.4
								Alcohol			Suicide Ideation	30	55.5
								Unspecified Substances	19	7.1	Suicide Attempt	7	36.84
								Unspecified Substances			Suicide Ideation	12	63.16
Kim et al. [12]	2022	South Korea	Previously hospitalized South Korean patients with self-harm episodes	388	≥18	Male: 65.5% Female: 34.5%	388	Alcohol	147	37.88	Suicide Attempt	147	100
Anand et al. [13]	2022	Poland	Hospitalized patients in Northern Poland treated for self-poisoning	634	18–76	Male: 40% Female: 60%	40	Alcohol	21	3.31	Suicide Attempt	21	100
Bismark Marie et al. [14]	2022	Australia	Australian frontline and allied health care workers surveyed mid-pandemic	7795	≥18	Male: 19% Female: 81%	819	Alcohol	2056	26.37	Suicide Ideation	309	15.02
Mamun et al. [15]	2021	Bangladesh	The general adult population in Bangladesh surveyed nationwide during the pandemic	10,067	20–39	Male: 56.1% Female: 43.9%	506	Tobacco	1486	14.76	Suicide Ideation	88	5.92
								Alcohol	267	2.65	Suicide Ideation	23	8.61
Goodyear et al. [16]	2021	Canada	LGBTQ2+ adults in Canada surveyed for mental health and substance use during COVID-19	502	≥18	Male: 55.6% Female: 39.6% Other: 4.8%	85	Alcohol	123	24.5	Suicide Ideation	32	26
								Cannabis	93	18.52	Suicide Ideation	36	38.7
Deimel D et al. [17]	2022	Germany	Volunteer German adults surveyed online during the first COVID-19 lockdown	2369	25–64	Male: 30.9% Female: 67.8% Other: 1–5%	938	Alcohol	623	26.3	Suicide Ideation	275	44.14
								Cannabis	96	03.01	Suicide Ideation	57	59.37
Nichter et al. [18]	2021	United States	U.S. military veterans enrolled in a longitudinal mental health study (pre- and during-COVID comparison)	3078	≥18	Male: 91.6% Female: 8.4%	241	Unspecified Substances	52	1.68	Suicide Ideation	52	100
Lardier et al. [19]	2022	United States	U.S. university students surveyed during Fall 2020 on alcohol use and mental health	606	18–25	Male: 38.8% Female: 61.2%	70	Alcohol	606	100	Suicide Ideation	62	10.23
Nomura et al. [20]	2021	Japan	University students from a Japanese national university during the May–June 2020 stay-at-home order	2449	18–22	Male: 54% Female: 45%	162	Alcohol	65	2.65	Suicide Ideation	48	73.84
Trettel, A. et al. [21]	2022	Brazil	Brazilian adults from the Amazonian Legal Region (10 municipalities) surveyed during COVID-19	4203	18–93	Female: 64.8% Male: 35.2%	3395	Alcohol	3074	73	Suicide Ideation	3074	100
								Tobacco	3214	76	Suicide Ideation	3214	100
								Unspecified Substances	1240	29.5	Suicide Ideation	1240	100
Varin, M et al. [22]	2022	Canada	Canadian adults nationally sampled in Fall 2020 (Statistics Canada) for alcohol and suicide ideation	12,344	≥18	Female: 57.1% Male: 42.9%	296	Alcohol	126	1.02	Suicide Ideation	94	74.6
de Moura PT. et al. [23]	2022	Brazil	Adults with substance use disorder receiving treatment at a psychosocial care center in Brazil	70	≥18	Female: 57.1% Male: 42.9%	29	Unspecified Substances	51	72.86	Suicide Risk	29	56.86
Caravaca-Sánchez F. et al. [24]	2022	Spain	Surveyed Spanish university students from a public university	517	18–50	Female: 86.4% Male: 13.6%	175	Alcohol	377	72.92	Suicide Risk	92	24.4
								Tobacco	169	32.68	Suicide Risk	54	31.95
								Cannabis	63	20.1	Suicide Risk	29	46.03
Maguire et al. [25]	2022	Ireland	Patients presenting with self-harm or suicidal ideation at an Irish emergency department	3749	18–60	Female: 50% Male: 50%	3749	Unspecified Substances	1156	30.83	Unspecific Suicide Behavior	1156	100
Otiñano et al. [26]	2022	Spain	Patients attended for suicidal behavior in a psychiatric emergency department	487	18–57	Female: 59.2% Male: 40.8%	487	Alcohol	24	4.93	Unspecific Suicide Behavior	24	100
								Unspecified Substances	56	11.5	Unspecific Suicide Behavior	56	100
Ridout K. et al. [27]	2021	United States	Adults (≥18 years) attended for suicide-related emergency department encounters at Kaiser Permanente Facility in Northern California	8190	≥18	Female 57.1% Male 42.9%	8190	Unspecified Substances	881	10.75	Unspecific Suicide Behavior	881	100
Vuscan M.E et al. [28]	2022	Romania	Suicide attempts and completed suicides from forensic and hospital data	170	Not reported	Not reported	170	Alcohol	17	10	Unspecific Suicide Behavior	17	100
								Unspecified Substances	2	31.1	Unspecific Suicide Behavior	2	100
Xu Y et al. [29]	2021	China	University students from 30 universities in Wuhan (Hubei Province)	11,254	≥15	Female: 63.98% Male: 36.02%	229	Alcohol	663	5.89	Unspecific Suicide Behavior	35	5.28
								Tobacco	424	3.77	Unspecific Suicide Behavior	16	3.77
	**Total**			**70,684**			**21,663**		**17,384**			**11,382**	

## Data Availability

The original contributions presented in this study are included in the article/Supplementary Material. Further inquiries can be directed to the corresponding author.

## Supplementary Materials

The following supporting information can be downloaded at: https://www.mdpi.com/article/10.3390/jcm15041328/s1, PRISMA 2020 Checklist.

## References

[1] Zvolensky M.J., Garey L., Rogers A.H., Schmidt N.B., Vujanovic A.A., Storch E.A., Buckner J.D., Paulus D.J., Alfano C., Smits J.A.J. (2020). Psychological, Addictive, and Health Behavior Implications of the COVID-19 Pandemic. Behav. Res. Ther..

[2] Ganesan B., Al-Jumaily A., Fong K.N.K., Prasad P., Meena S.K., Tong R.K.-Y. (2021). Impact of Coronavirus Disease 2019 (COVID-19) Outbreak Quarantine, Isolation, and Lockdown Policies on Mental Health and Suicide. Front. Psychiatry.

[3] Vogel E.A., Chieng A., Robinson A., Pajarito S., Prochaska J.J. (2021). Associations Between Substance Use Problems and Stress During COVID-19. J. Stud. Alcohol Drugs.

[4] Sinha R. (2001). How Does Stress Increase Risk of Drug Abuse and Relapse?. Psychopharmacology.

[5] Singh S., Mani Pandey N., Datta M., Batra S. (2021). Stress, Internet Use, Substance Use and Coping among Adolescents, Young-Adults and Middle-Age Adults amid the ‘New Normal’ Pandemic Era. Clin. Epidemiol. Glob. Health.

[6] Poorolajal J., Haghtalab T., Farhadi M., Darvishi N. (2016). Substance Use Disorder and Risk of Suicidal Ideation, Suicide Attempt and Suicide Death: A Meta-Analysis. J. Public Health.

[7] Borges G., Bagge C.L., Cherpitel C.J., Conner K.R., Orozco R., Rossow I. (2016). A Meta-Analysis of Acute Alcohol Use and the Risk of Suicide Attempt. Psychol. Med..

[8] de Holanda Júnior W.P., Maceno R.H.M., Ferreira M.A.D. (2024). Sociodemographic Factors of Violent Deaths Related to Licit or Ilicit Psychoactive Substances: A Cross-Sectional Study, Ceará, Brazil, 2015–2019. Epidemiol. Serv. Saúde.

[9] Kang J., Lim J., Lee J., Shin J.-Y. (2024). Suicide Rates and Subgroups With Elevated Suicide Risk Among Patients With Psychiatric Disorders: A Nationwide Cohort Study in Korea. J. Korean Med. Sci..

[10] Gerstner R.M., Narváez F., Leske S., Troya M.I., Analuisa-Aguilar P., Spittal M.J., Gunnell D. (2022). Police-Reported Suicides during the First 16 Months of the COVID-19 Pandemic in Ecuador: A Time-Series Analysis of Trends and Risk Factors until June 2021. Lancet Reg. Health Am..

[11] Bukuluki P., Wandiembe S., Kisaakye P., Besigwa S., Kasirye R. (2021). Suicidal Ideations and Attempts Among Adolescents in Kampala Urban Settlements in Uganda: A Case Study of Adolescents Receiving Care From the Uganda Youth Development Link. Front. Sociol..

[12] Kim M.-J., Paek S.-H., Kwon J.-H., Park S.-H., Chung H.-J., Kim M.-J., Paek S.-H., Kwon J.-H., Park S.-H., Chung H.-J. (2022). Changes in Suicide Rate and Characteristics According to Age of Suicide Attempters before and after COVID-19. Children.

[13] Anand Ł.S., Anand J.S. (2022). Self-Poisonings before and during the Initial Year of the COVID-19 Pandemic in Northern Poland. Int. J. Occup. Med. Environ. Health.

[14] Bismark M., Scurrah K., Pascoe A., Willis K., Jain R., Smallwood N. (2022). Thoughts of Suicide or Self-Harm among Australian Healthcare Workers during the COVID-19 Pandemic. Aust. N. Z. J. Psychiatry.

[15] Mamun M.A., Sakib N., Gozal D., Bhuiyan A.I., Hossain S., Bodrud-Doza M., Al Mamun F., Hosen I., Safiq M.B., Abdullah A.H. (2021). The COVID-19 Pandemic and Serious Psychological Consequences in Bangladesh: A Population-Based Nationwide Study. J. Affect. Disord..

[16] Goodyear T., Slemon A., Richardson C., Gadermann A., Salway T., Dhari S., Knight R., Jenkins E. (2021). Increases in Alcohol and Cannabis Use Associated with Deteriorating Mental Health among LGBTQ2+ Adults in the Context of COVID-19: A Repeated Cross-Sectional Study in Canada, 2020–2021. Int. J. Environ. Res. Public Health.

[17] Deimel D., Firk C., Stöver H., Hees N., Scherbaum N., Fleißner S. (2022). Substance Use and Mental Health during the First COVID-19 Lockdown in Germany: Results of a Cross-Sectional Survey. Int. J. Environ. Res. Public Health.

[18] Nichter B., Hill M.L., Na P.J., Kline A.C., Norman S.B., Krystal J.H., Southwick S.M., Pietrzak R.H. (2021). Prevalence and Trends in Suicidal Behavior Among US Military Veterans During the COVID-19 Pandemic. JAMA Psychiatry.

[19] Lardier D.T., Zuhl M.N., Holladay K.R., Amorim F.T., Heggenberger R., Coakley K.E. (2023). A Latent Class Analysis of Mental Health Severity and Alcohol Consumption: Associations with COVID-19-Related Quarantining, Isolation, Suicidal Ideations, and Physical Activity. Int. J. Ment. Health Addict..

[20] Nomura K., Minamizono S., Maeda E., Kim R., Iwata T., Hirayama J., Ono K., Fushimi M., Goto T., Mishima K. (2021). Cross-Sectional Survey of Depressive Symptoms and Suicide-Related Ideation at a Japanese National University during the COVID-19 Stay-Home Order. Environ. Health Prev. Med..

[21] Trettel A.C.P.T., Muraro A.P., de Oliveira E.C., Do Nascimento V.F., Andrade A.C.D.S., Dos Santos E.S., Espinosa M.M., Pillon S.C. (2022). Factors Associated with Suicidal Ideation during the COVID-19 Pandemic in a Population in the Brazilian Legal Amazon. Ciênc. Saúde Coletiva.

[22] Varin M., Liu L., Gabrys R., Gariepy G., MacEachern K.H., Weeks M. (2023). Increased Alcohol Use, Heavy Episodic Drinking, and Suicide Ideation during the COVID-19 Pandemic in Canada. Can. J. Public Health.

[23] de Moura P.T., Rockenbach C.A., Mendes C.D.R., Mendes G.U., Ghiggi L.A., Diel M., Martini P., Camozzato P.F., de Castro R.S.B., Mello de Mello R. (2022). Depression and Suicide Risk during the Covid-19 Pandemic at a Brazilian Public Health Psychosocial Addiction Care Center: A Preliminary Report. Trends Psychiatry Psychother..

[24] Caravaca-Sánchez F., Muyor-Rodríguez J., Fernández-Prados J.S. (2022). Risk and Protective Factors Associated with Suicidal Behaviour during the COVID-19 Pandemic Crisis amongst College Students in Spain. Soc. Work Ment. Health.

[25] Maguire E., Kavalidou K., Bannan N., Doherty A.M., Jeffers A. (2022). Substance Use and Self-Harm Emergency Department Presentations during COVID19: Evidence from a National Clinical Programme for Self-Harm. Ir. J. Psychol. Med..

[26] Irigoyen-Otiñano M., Nicolau-Subires E., González-Pinto A., Adrados-Pérez M., Buil-Reiné E., Ibarra-Pertusa L., Albert-Porcar C., Arenas-Pijoan L., Sánchez-Cazalilla M., Torterolo G. (2022). Characteristics of Patients Treated for Suicidal Behavior during the Pandemic in a Psychiatric Emergency Department in a Spanish Province. Rev. Psiquiatr. Salud Ment..

[27] Ridout K.K., Alavi M., Ridout S.J., Koshy M.T., Awsare S., Harris B., Vinson D.R., Weisner C.M., Sterling S., Iturralde E. (2021). Adult Suicide-Related Emergency Department Encounters during the COVID-19 Pandemic: A Cross-Sectional Study. Lancet Reg. Health—Am..

[28] Vuscan M.E., Buciuta A., Vica M.L., Balici S., Rusu S.I., Siserman C.V., Coman H.G., Matei H.V. (2023). Impact of the COVID-19 Pandemic on the Suicidal Behavior in Romania. Arch. Suicide Res..

[29] Xu Y., Su S., Jiang Z., Guo S., Lu Q., Liu L., Zhao Y., Wu P., Que J., Shi L. (2021). Prevalence and Risk Factors of Mental Health Symptoms and Suicidal Behavior Among University Students in Wuhan, China During the COVID-19 Pandemic. Front. Psychiatry.

[30] Conner K.R., Bridge J.A., Davidson D.J., Pilcher C., Brent D.A. (2019). Metaanalysis of Mood and Substance Use Disorders in Proximal Risk for Suicide Deaths. Suicide Life Threat. Behav..

[31] Bindu R., Sharma M.K., Suman L.N., Marimuthu P. (2011). Stress and Coping Behaviors among Smokers. Asian J. Psychiatry.

[32] Leung C.M.C., Ho M.K., Bharwani A.A., Cogo-Moreira H., Wang Y., Chow M.S.C., Fan X., Galea S., Leung G.M., Ni M.Y. (2022). Mental Disorders Following COVID-19 and Other Epidemics: A Systematic Review and Meta-Analysis. Transl. Psychiatry.

[33] Reinke M., Falke C., Cohen K., Anderson D., Cullen K.R., Nielson J.L. (2023). Increased Suicidal Ideation and Suicide Attempts in COVID-19 Patients in the United States: Statistics from a Large National Insurance Billing Database. Psychiatry Res..

