# Supplementary material for: Relationship Between Substance Use and Suicide Behavior During the COVID-19 Pandemic: A Systematic Review and Random-Effects Proportions Meta-Analysis
Source: J Clin Med. 2026 Feb 7;15(4):1328. doi: 10.3390/jcm15041328 (PMC12942258; doi:10.3390/jcm15041328)
Supplement: Supplementary file 1 [file jcm-15-01328-s001.zip › jcm-4089258-supplementary.pdf]

PRISMA 2020 Checklist

Section and Topic	Item #	Checklist item	Location reported in manuscript
Title	1	Identify the report as a systematic review	Title (p. 1)
Abstract	2	Structured abstract	Abstract (p. 1)
Introduction – Rationale	3	Rationale in context	Introduction, paragraphs 1–3 (pp. 2–3)
Introduction – Objectives	4	Objectives or questions	End of Introduction (pp. 2–3)
Methods – Eligibility criteria	5	Inclusion and exclusion criteria	Methods: Eligibility criteria (pp. 3–6)
Methods – Information sources	6	Databases and dates	Methods: Information Sources (pp. 3–6)
Methods – Search strategy	7	Full search strategy	Methods: Information Sources and Search Strategy (pp. 3–6)
Methods – Selection process	8	Study selection process	Methods: Selection process (pp. 3–6)
Methods – Data collection	9	Data extraction methods	Methods: Data Items (pp. 3–6)
Methods – Data items	10a	Outcomes defined	Methods: Data synthesis and analysis (pp. 3–6)
Methods – Other variables	10b	Other variables	Methods: Data Items (pp. 3–6)
Methods – Risk of bias	11	Risk of bias tools	Methods: Risk of bias (pp. 3–6)
Methods – Effect measures	12	Effect measures	Methods: Data synthesis and analysis (pp. 3–6)
Methods – Synthesis methods	13a–f	Synthesis and meta-analysis methods	Methods: Data synthesis and analysis (pp. 3–6)
Methods – Reporting bias	14	Reporting bias assessment	Methods: Data synthesis and analysis (pp. 3–6)
Methods – Certainty assessment	15	Certainty of evidence	Not formally assessed
Results – Study selection	16a	Search results	Results: Study Selection; Figure 1 (pp. 6–11) (pp. 7–10)
Results – Excluded studies	16b	Excluded studies	Results: Study Selection (pp. 6–11)
Results – Study characteristics	17	Study characteristics	Results; Table 1 (pp. 6–11) (pp. 8–9)

Results – Risk of bias	18	Risk of bias results	Results: Risk of Bias (pp. 6–11)
Results – Individual studies	19	Individual study results	Results; Table 1 (pp. 6–11) (pp. 8–9)
Results – Syntheses	20a–d	Meta-analysis results	Results; Figures 3–5 (pp. 6–11) (pp. 7–10)
Results – Reporting bias	21	Reporting bias results	Qualitative assessment in Results (pp. 6–11)
Results – Certainty	22	Certainty of evidence	Not formally assessed
Discussion	23a–d	Interpretation, limitations, implications	Discussion (pp. 11–14)
Other – Registration	24a	PROSPERO registration	Methods: Registration (pp. 3–6)
Other – Protocol	24b	Protocol availability	Methods (pp. 3–6)
Other – Amendments	24c	Protocol amendments	Not applicable
Other – Support	25	Funding sources	Author Contributions
Other – Competing interests	26	Conflicts of interest	Conflicts of Interest
Other – Data availability	27	Data availability	End of manuscript
